# One-Pot Hydrothermal Synthesis of Victoria Green (Ca_3_Cr_2_Si_3_O_12_) Nanoparticles in Alkaline Fluids and Its Colour Hue Characterisation

**DOI:** 10.3390/nano11020521

**Published:** 2021-02-18

**Authors:** Juan Carlos Rendón-Angeles, Zully Matamoros-Veloza, Jose Luis Rodríguez-Galicia, Gimyeong Seong, Kazumichi Yanagisawa, Aitana Tamayo, Juan Rubio, Lluvia A. Anaya-Chavira

**Affiliations:** 1 Centre for Research and Advanced Studies of the National Polytechnic Institute, Saltillo Campus, Ramos Arizpe, Coahuila 25900, Mexico; jose.rodriguez@cinvestav.edu.mx (J.L.R.-G.); lluvia.anaya@cinvestav.mx (L.A.A.-C.); 2 Tecnológico Nacional de México/(I.T. Saltillo), Technological Institute of Saltillo, Graduate Division, Saltillo 25280, Mexico; zully.mv@saltillo.tecnm.mx; 3 New Industry Creation Hatchery Center, Tohoku University, 6-6-10 Aoba, Aramaki, Aoba-Ku, Sendai 980-8579, Japan; kimei.sei.c6@tohoku.ac.jp; 4 Research Laboratory of Hydrothermal Chemistry, Faculty of Science, Kochi University, Kochi 780-8073, Japan; yanagi@kochi-u.ac.jp; 5 Institute of Ceramics and Glass, CSIC, 28049 Madrid, Spain; aitanath@icv.csic.es (A.T.); jrubio@icv.csic.es (J.R.)

**Keywords:** Ca_3_Cr_2_Si_3_O_12_, Victorian green pigment, hydrothermal synthesis, one-pot reaction, hierarchical architecture, nanosized dyes

## Abstract

One-pot hydrothermal preparation of Ca_3_Cr_2_Si_3_O_12_ uvarovite nanoparticles under alkaline conditions was investigated for the first time. The experimental parameters selected for the study considered the concentration of the KOH solvent solution (0.01 to 5.0 M), the agitation of the autoclave (50 rpm), and the nominal content of Si^4+^ (2.2–3.0 mole). Fine uvarovite particles were synthesised at 200 °C after a 3 h interval in a highly concentrated 5.0 M KOH solution. The crystallisation of single-phase Ca_3_Cr_2_Si_3_O_12_ particles proceeded free of by-products via a one-pot process involving a single-step reaction. KOH solutions below 2.5 M and water hindered the crystallisation of the Ca_3_Cr_2_Si_3_O_12_ particles. The hydrothermal treatments carried out with stirring (50 rpm) and non-stirring triggered the crystallisation of irregular anhedral particles with average sizes of 8.05 and 12.25 nm, respectively. These particles spontaneously assembled into popcorn-shaped agglomerates with sizes varying from 66 to 156 nm. All the powders prepared by the present method exhibited CIE-L*a*b* values that correspond to the Victoria green colour spectral space and have a high near infrared reflectance property. The particle size and structural crystallinity are factors affecting the Victoria pigment optical properties, such as CIE-L*a*b* values, green tonality, and near-infrared reflectance.

## 1. Introduction

The traditional garnet structured Ca_3_Cr_2_Si_3_O_12_, commercially known as Victorian green, has been used as the source of colour in paintings and decoration, amongst other applications. This inorganic pigment had been available in the Earth’s crust as a mineral, as the green garnet uvarovite, but only a limited number of green mineral pigments were accessible [1,2,3]. The uvarovite garnet is a member of a broad group of mineral silicates containing Ca^2+^ and Cr^3+^ ions as crystalline structural constituents. The calcic varieties are formed on the earth crust at lower pressures but also synthetically [3,4]. The mineral uvarovite has a dark-green tonality, and it belongs to the crystalline cubic structure (space group *Ia-3d,* No. 230) with unit cell 11.5–12.5 Å with chemical formula Ca_3_Cr_2_Si_3_O_12_ [4,5,6,7,8]. The silicate garnet crystalline structure consists of a homogeneous distributed corner-sharing SiO_4_ tetrahedral units and CrO_6_, together with twisted CaO_8_ cubes allocated in between. The Cr^3+^ ions forming the CrO_6_ octahedra unit is the chromophore that produces the dark-green hue. This particular garnet and its solid solutions have been the research subject due to its optical, magnetic, and electrical properties [3,6].

The pioneering work aiming at the preparation of the uvarovite pigment powders was conducted in 1885 with the Victorian green pigment powder obtained by solid-state reaction at high temperature 1000–1370 °C using a powder mixture containing K_2_Cr_2_O_7_, CaCO_3_, and SiO_2_ [9]. Various research works have been conducted since the 1950s. Efforts have been devoted to improving the solid-state reaction synthesis conditions, focusing on the raw precursor selection and the treatment temperature (855–1490 °C) [9,10,11,12]. In addition, the Cr^3+^ loss caused by Cr_2_O_3_ high volatility above 1200 °C, coupled with the electric valence change of chromium (Cr^2+^, Cr^3+^, Cr^6+^, and occasionally Cr^4+^ and Cr^5+^) are other processing disadvantages of this method. These factors are crucial to control the chemical compositional homogeneity of uvarovite garnets [12,13]. More recently, soft chemical processing (sol–gel), coupled with a calcination stage, has been under exhaustive investigation. This process had favoured the preparation of uvarovite and its solid solutions at even lower temperatures ranging from 700 to 900 °C in air atmosphere [13,14,15].

However, among the soft chemical methods available for inorganic material production, hydrothermal processing has delivered high processing efficiency for the preparation of nano-sized inorganic silicate pigments. In particular, pyroxene silicates, namely XYSi_2_O_6_ (where X and Y are both divalent metal cations), were successfully crystallised under mildly alkaline hydrothermal conditions between 180 and 240 °C [16]. The crystallisation of 3D hierarchical morphologies occurred by transforming the precursor colloidal gel by a controlled dissolution–crystallisation mechanism. The tuning of the colour tonality of BaCuSi_2_O_6_ violet stain occurred preferentially in alkaline hydrothermal media. The colour hue control was promoted by the use of NO_3_^−^ anions, which controlled the nanometric size particle assembly process to form the fine nest-like shaped particles [16]. A literature survey suggests that natural green uvarovite crystals were likely formed under hydrothermal conditions in the Earth’s crust. On the contrary, the crystallisation of the uvarovite garnet species has not been yet investigated in alkaline hydrothermal media under controlled laboratory conditions, which is the main subject in the present study.

In the work reported here, the efforts were devoted to investigating the optimum hydrothermal reaction conditions for the crystallisation of Victorian green pigment (Ca_3_Cr_2_Si_3_O_12_). In particular, we have systematically investigated the effect of various critical processing parameters that might trigger the crystallisation of the single-phase Ca_3_Cr_2_Si_3_O_12_ and the control of particle growth and morphology. The alkaline hydrothermal media concentration, stirring of the mother liquor, and the nominal mixed Si^4+^ molar content were the main parameters varied. Hypothetically, the use of KOH solutions coupled with the control of the Si^4+^ ions content deficiency might lead to promoting controlled hydrothermal conditions that would hinder the oxidation of Cr^3+^ to other valences (Cr^4+^ or Cr^6+^). This approach might lead to the fine-tuning of a one-pot hydrothermal process for preparing nanosized Ca_3_Cr_2_Si_3_O_12_ particles free of reaction by-products. Likewise, the control of the Ca_3_Cr_2_Si_3_O_12_ particle morphology found in the precursor co-precipitated gel containing a Si^4+^ deficiency in alkaline solution could result in the tuning of the green tonalities of the pigment; the effectiveness of these approaches is discussed in detail below.

## 2. Materials and Methods

### 2.1. Materials and Ca:Cr:Si Precursor Preparation

Hydrated chemical-grade reagents (Wako, Osaka, Japan, 99.0% purity) calcium nitrate (Ca(NO_3_)_2_·4H_2_O), chromium nitrate (Cr(NO_3_)_3_·9H_2_O), and sodium metasilicate (Na_2_SiO_3_·9H_2_O) were used for the precursor gel preparation. Metal precursor solutions were prepared with deionised water at a concentration of 0.375 M for Ca(NO_3_)_2_ and Na_2_SiO_3_, and 0.25 M for Cr(NO_3_)_3_. The concentration of the KOH solutions selected varied in the range of 0.01–5.0 M. Equivalent volume solution mixing Ca:Cr:Si ratios of 5.5:5.5:5.5 mL (16.5 mL) and 7.5:7.5:7.5 mL (22.5 mL) were selected. Theses solution mixture ratios provide the Victorian green pigment stoichiometric Ca:Cr:Si molar ratio of 3:2:3. Initially, the Ca and Cr solutions were mixed in the Teflon chamber bottom; then, the Na_2_SiO_3_ solution was poured, causing spontaneously the precipitation of a milky light green colour (Gel Ca:Cr:Si) colloid. The colloidal precursor is made up of an amorphous phase and crystalline SiO_2_ as shown in the X-ray diffraction plots in the Section 3.1. Subsequently, the KOH solution volume (12.5 or 18.5 mL) was added, all the experiments were carried at a total mother liquor solution volume of 35 mL with the autoclave 50% full.

### 2.2. Hydrothermal Treatments

#### 2.2.1. Treatments Conducted under Stirring Conditions

**Figure 1:**
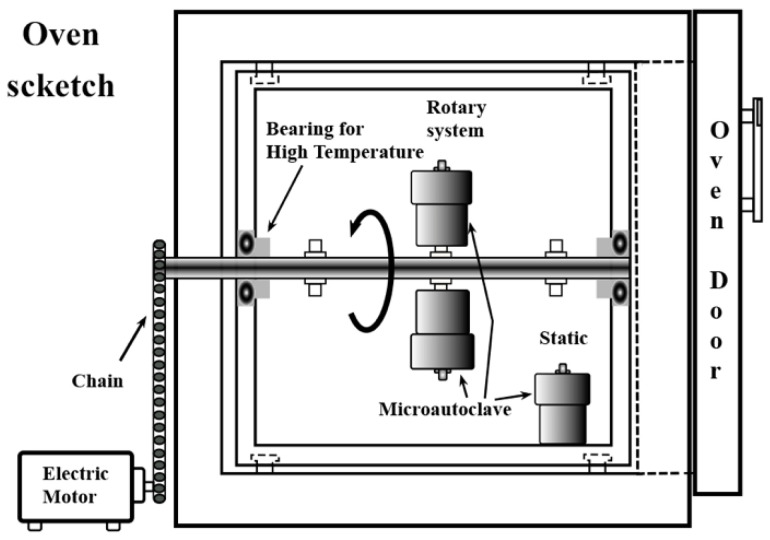
Scheme of the experimental oven used for carrying out the hydrothermal treatments under stirring and static conditions.

The selected parameters to investigate the Victorian green pigment synthesis are as follows: the micro-autoclave agitation speed, the alkaline media concentration, and the KOH solution volume. The study also includes evaluating the effect of the kinetic parameters (temperature and reaction time). These experiments aimed to determine the effect of the alkalinity and the stirring of the hydrothermal medium to produce the Ca_3_Cr_2_Si_3_O_12_ phase. After mixing the precursor solutions, a volume of 18.5 mL of KOH solution with concentrations between 0 and 5.0 M was added. The autoclaves were sealed and placed in the rotatory device assembled within the convection oven (Figure 1). Then, the vessels were heated at a constant 5 °C speed to reach 220 °C, and the treatments were conducted for 24 h at a constant stirring speed of 50 rpm.

#### 2.2.2. Treatments Conducted under Non-Stirring Conditions

The second experimental set was conducted to evaluate the feasibility for promoting the one-pot reaction without stirring. The treatments were carried using a 5.0 M KOH solution with two volumes (12.5 and 18.5 mL) for different reaction intervals (3–72 h) and temperatures (200–240 °C). Additionally, some experiments were conducted with different Si^4+^ molar contents (2.2–2.8 mole). According to the nominal 3.0 mole Si^4+^ (7.5 mL), the lower contents of 2.2, 2.4, 2.6, and 2.8 Si^4+^ mole were added proportionally to the volume ratio Si^4+^:H_2_O of 5.5:2.0, 6.0:1.5, 6.5:1.0, and 7.0:0.5, respectively. After the solution mixing, the autoclave vessel was sealed and heated inside a convection oven to reach the desired temperature. After each treatment, the reaction products were gravimetrically separated and then washed vigorously, four times, with hot water (60 °C) to clean the remaining alkaline medium. Then, the resultant powders were dried overnight in an oven at 80 °C.

### 2.3. Characterisation

Powder X-ray diffraction (PXRD). The analyses were conducted in a Rigaku Ultima IV diffractometer (Akishima, Tokyo, Japan) operated at 40 kV and 20 mA, using Cu-Kα radiation (*λ* = 1.54056 Å). XRD analyses were collected in the 2θ range of 5–80° at a constant scanning speed of 20°/min with a 0.02° step. Rietveld refinement analyses were carried out on the diffraction patterns collected in the 2θ range of 15–130°, under standard conditions at a scanning speed of 0.01°/min and 0.002° step. The refinement calculations were performed using TOPAS 4.2 (Bruker AXS: Karlsruhe, Germany, 2009) software. The space group, together with the atomic position (Wyckoff number and coordinates) correspond to the COD card 96-900-7150. Additional details associated with the Rietveld refinement analysis are given in the Supplementary Supporting Information File, Section S2.3.

Fourier transform infrared spectroscopy (FT-IR). Additional structural details associated with the presence of water molecules were investigated using a FT-IR apparatus JASCO 4000, Hachioji, Tokyo, Japan. The analyses were conducted using pelletised samples prepared with 5 mg of pigment and 200 mg KBr. The powder samples were dried overnight at 60 °C previously the pellet preparation.

Morphology and microstructural observations. The microstructural aspects of the Ca_3_Cr_2_Si_3_O_12_ particles were observed by (FE-SEM JEOL JSM-7100F, Akishima, Tokyo, Japan) operated at 10 kV and 69 μA. The particle size distribution was measured from SEM images of 50 particles. Crystalline structural details of Victoria pigment particles were revealed by (HR-TEM, Philips Titan 300) operated at 300 kV.

Differential thermal analysis (DTA). Ca_3_Cr_2_Si_3_O_12_ pigment thermal stability was evaluated via thermogravimetric and differential thermal analysis (Perkin Elmer Pyris Diamond TG/DTA, Waltham, MA, USA) from 30 to 1000 °C. The analyses were carried at 10 °C/min constant heating rate in air atmosphere corresponding to the apparatus furnace chamber volume.

Optical properties. The Victoria green powders optical properties, colour CIEL*a*b*, and reflectance spectra were measured in a UV-vis near infrared (NIR) spectrometer (Perkin-Elmer Lambda 25, 800–2500 nm, Waltham, MA, USA). The BaSO_4_ fine powder was used to calibrate the colour space parameters, as suggested by the standard CIE-L*a*b* colourimetry method.

## 3. Results and Discussion

A proposed one-pot hydrothermal processing scheme was investigated as a new and potentially feasible route for preparing synthetic Victorian green pigment particles (Ca_3_Cr_2_Si_3_O_12_). Initial effort was directed toward establishing the appropriate alkaline concentration over the range 0.0–5.0 M that triggers the crystallisation of Ca_3_Cr_2_Si_3_O_12_, since this had not yet been found for this pigment in hydrothermal fluids. Table 1 summarises the conditions of the selected experiments conducted in various KOH solutions that produced Ca_3_Cr_2_Si_3_O_12_ particles under alkaline hydrothermal conditions with interesting green tonalities. The major crystalline phases identified are also included together with the amount of the secondary phases calculated by the Rietveld refinement.

### 3.1. The Effect of the Alkalinity on the Ca_3_Cr_2_Si_3_O_12_ Chemical Stability under Hydrothermal Synthesis

**Figure 2:**
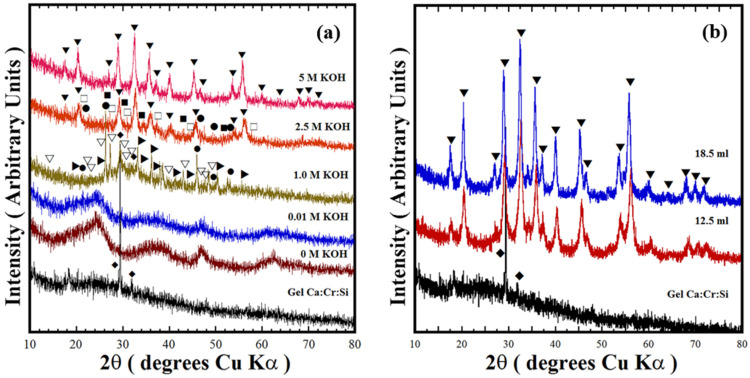
XRD patterns of the reaction products produced at 220 °C for 24 h with different KOH solvent solution (18.5 mL) concentrations. The experiments were carried out with stoichiometrically mixed Si^4+^ content of 3.0 mole (**a**). Those (**b**) of the powders prepared with two different volumes of the 5.0 M KOH solution; these experiments were all conducted at an autoclave stirring speed of 50 rpm. Indexed crystalline phases: (▼) Uvarovite structure Ca_3_Cr_2_Si_3_O_12_, COD card no. 96-900-7150, crystalline secondary phases: (♦) SiO_2_, (●) SiO_2_ Quartz high COD card 96-101-1201, (▽) Ca_2_SiO_4_ COD card no. 96-210-3317, (▶) CaCr_2_O_4_ COD card no. 96-200-2211 phase, (■) SiO_2_ Coesite COD card no. 96-900-0805, (☐) Ca_2_SiO_5_ COD card 96-200-1356.

Figure 2 shows the typical XRD patterns of residual powders produced at 240 °C for 24 h under stirring at 50 rpm, employing water and various KOH alkaline solutions (0–5.0 M). These experiments aimed to determine the potential feasibility to produce Ca_3_Cr_2_Si_3_O_12_ uvarovite-structured in alkaline hydrothermal medium for the first time. Generally, the synthesis of Ca_3_Cr_2_Si_3_O_12_ particles did not occur when water and low concentrated KOH (0.01–0.05 M) solutions were used as hydrothermal medium. The PXRD pattern of the reaction products could not be indexed with analogous inorganic compounds in the CaO–Cr_2_O_3_–SiO_2_ system. On the other hand, using KOH media with concentrations of 0.5 and 1.0 M resulted in the formation of secondary phases of SiO_2_ (Quartz high (●)), Ca_2_SiO_4_ (▽), and CaCr_2_O_4_ (▶). The Ca:Cr:Si gel chemical reactivity was enhanced in a mildly concentrated 2.5 M KOH solution, resulting in the formation of Ca_3_Cr_2_Si_3_O_12_ (69.63 ± 2.5 wt %) together with SiO_2_ (Quartz high (●), Coesite (■)) and Ca_2_SiO_5_ (☐) crystalline phases. The Rietveld refinement algorithm calculated the content of each phase identified, and the schematic results are shown in Figure S1c,d (Supplementary Supporting Data File).

The results show that Ca_3_Cr_2_Si_3_O_12_ single-phase free of reaction by-products occurs only in highly concentrated KOH media (5.0 M). The X-ray pattern of this sample was indexed with that of the cubic crystalline phase (COD Card No. 96-900-7150, space group *Ia-3d*, No. 230 (▼)), as seen in Figure 2. Likewise, the KOH nominal volume did not hinder the crystallisation of Ca_3_Cr_2_Si_3_O_12_; this compound solely was obtained either of the 5.0 M KOH solution of 12.5 mL (Sample ID CCS2) or 18.5 mL (Sample ID CCS1); see Figure 2b. These results bear evidence indicating that the one-pot hydrothermal reaction occurs in a single-step chemical reaction and is preferentially triggered under strongly alkaline conditions. The concentrated alkaline media dissolves the amorphous colloid precursor and the SiO_2_, and as a consequence, solute supersaturation is reached in the system and it achieves the chemical equilibrium associated with Equation (1). The chemical reaction (Equation (1)) is conducive to Victorian green pigment powder crystallisation under the proposed one-pot hydrothermal reaction. It deserves emphasising that based on the reaction pathway elucidated by the XRD results, we infer that the KOH media is crucial to mitigate the acidic capability the anionic species, namely the high oxidant NO^3−^ ions, which might trigger the valence oxidation of Cr^3+^ to Cr^6+^. The reaction trend is similar to that determined on silicate pigments, BaCuSi_2_O_6_ [16] and BaCu_2_Si_2_O_7_ [17], where the reduction of Cu^2+^ to Cu^+^ did not take place under alkaline hydrothermal conditions. Chromium oxidation might hinder the Ca_3_Cr_2_Si_3_O_12_ crystallisation due to the formation of more stable crystalline phases containing chromium (VI). This processing advantage is one of the factors associated to the one-pot hydrothermal processing efficiency in comparison with the solid-state reaction method widely used to prepare the Victorian green pigment [9,10,11,12,13]. Additionally, small crystalline structural differences were determined on the experiments carried at various temperatures and reaction intervals in 5 M KOH solution under hydrothermal treatments conducted without stirring. These results are reported in the Supplementary Supporting Information Data File, Section S3.

Ca_3_Cr_2_Si_3−y_(OH)_24−y(gel)_ + ySiO_2(s)_ + 5NO_3_^−^_(aq)_ + 2Na^+^_(aq)_ Ca_3_Cr_2_Si_3_O_12(s)_ + 5NO_3_^−^_(aq)_ + 2Na^+^_(aq)_ + (24 − y)OH^−^_(aq)_ (1)

### 3.2. Structural Features of the Victorian Green Ca_3_Cr_2_Si_3_O_12_ Powders Prepared under Alkaline Hydrothermal Conditions

The crystalline structural features of the cubic uvarovite particles prepared under the relevant treatment conditions are given in Table 1. Table 1 shows the results calculated by the Rietveld refinement analyses of parameters such as crystallite size, unit cell parameters, lattice strain, residual parameter *R_wp_* (%) and goodness-of-fit (GOF) (χ^2^) fitting refinement parameters. The refinement parameters are conducive of sufficient accuracy for calculating the structural features of the Victorian green pigment samples. The values of the *R_wp_* and goodness-of-fit factor (GOF) (χ^2^) (Table 1) are consistent with the Ca_3_Cr_2_Si_3_O_12_ residual line for the Victorian green pigment in the Rietveld refinement plots (Figure S2); these results reveal the high accuracy of the refinement approach used, leading to small residual differences between the experimental and calculated XRD patterns. The plots of selected pigments are shown in the Supporting Supplementary Information (see Figure S2). The unit cell lattice parameters of Ca_3_Cr_2_Si_3_O_12_ calculated are within the broad range of the “*a*_0_” lattice parameter, 11.5–12.5 Å, which is determined in various uvarovite minerals and its synthetic parents [4,5,6,7,8]. Interestingly, the “*a*_0_” lattice parameter varied within the range 12.2429–12.3372 Å (see Table 1) on the Victorian pigment powders. However, this variation is likely associated with the residual lattice strain induced in the crystallisation process as shown in Table 1, for the samples treated for short reaction intervals. However, the partial incorporation of water molecules in the Ca_3_Cr_2_Si_3_O_12_ under the one-pot hydrothermal process is another factor that might provoke the large lattice parameter values [4,18,19]. Natural mineral uvarovite is amongst the species formed at the upper mantle and transition zone. It usually incorporates water molecules that partially substitute the SiO_4_ tetrahedra units by OH^−^ in the form of H_4_O_4_ [1,18,19]. Hence, the residual lattice strain coupled with the presence of H_4_O_4_ caused the crystalline structural variation in the Victorian green pigments produced by the new route investigated.

**Figure 3:**
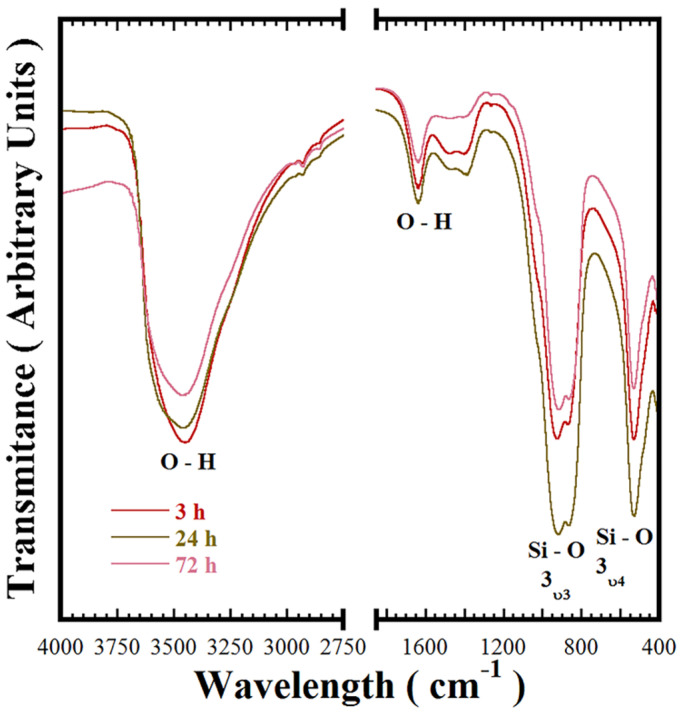
Fourier transform infrared (FT-IR) spectrum of Ca_3_Cr_2_Si_3_O_12_ pigment samples hydrothermally crystallised at 200 °C using a 5.0 M KOH solution (12.5 mL), the stoichiometric mixed Si^4+^ content used was 3.0 mole, and all the treatments were conducted without stirring for various reaction intervals as shown.

Additionally, Ca_3_Cr_2_Si_3_O_12_ crystalline features were also studied using FT-IR spectroscopic analyses over the wavenumber range of 400–4000 cm^−1^. These analyses were conducted to reveal additional features of the chemical bonds in the Ca_3_Cr_2_Si_3_O_12_ pigment. The FT-IR spectra of the samples prepared at 240 °C in the 5.0 M KOH solution (with a nominal Si^4+^ content of 3.0 mole) for various intervals are shown in Figure 3. Generally, the FT-IR results revealed no further chemical compositional differences between the samples because the same bands were observed irrespective of the reaction interval. Thus, the sharp band at 528.7 cm^−1^ is assigned to the Si-O bond symmetric bending mode (3*v*_4_), while two overlapped peaks at 861.1 and 920.8 cm^−1^ constituted the broad peak in the wavelength range of 750–1200 cm^−1^. These bands correspond to the asymmetric Si-O stretching mode (3*v*_3_) bands of the SiO_4_ units. The new signals at 1389.6, 1483.4, and 1636.9 cm^−1^, together with the broad one between 3000 and 3700 cm^−1^, are attributed to the presence of O-H bonding. These bands correspond to water molecules absorbed on the pigment powders. The present results provide clear confirmatory evidence of hydro-garnet formation in highly saturated alkaline hydrothermal media, which has not previously been reported [1,18,19]. Based on these results, we argued that the water molecules absorption occurs in the OH^−^ supersaturated media, due to OH^−^ accelerating the dissolution–crystallisation mechanism that transforms the precursor gel into the Ca_3_Cr_2_Si_3_O_12_. Water absorption onto the uvarovite particles surface is likely promoted via a hydro-substitution mechanism [18]. The water (wt %) content determined by thermal gravimetric analyses was 17.0 ± 2.5 wt %; this content was measured with various uvarovite powders prepared under different conditions as shown in Figure S4 (Supplementary Supporting Data). Furthermore, the water absorption is likely to provoke the Si-O band displacement in all the samples and induce the residual strain calculated by the Rietveld refinement analyses that disturb the lattice arrangement in the cubic uvarovite structure.

### 3.3. Tailoring the Synthesis of the Ca_3_Cr_2_Si_3_O_12_ by Controlling the Nominal Si^4+^ Content

**Figure 4:**
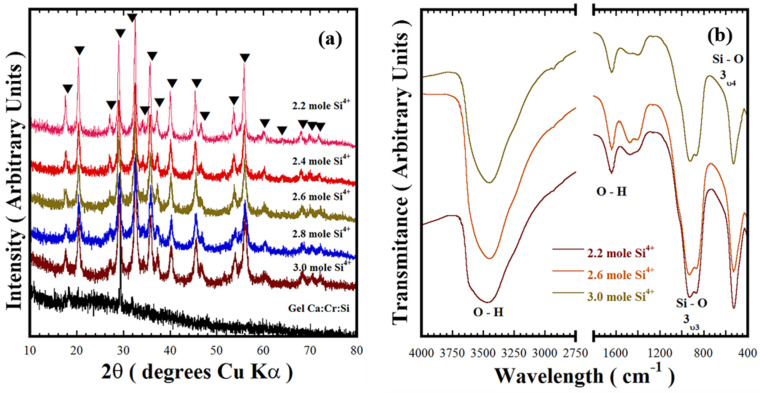
(**a**) XRD patterns and (**b**) FT-IR spectrum of the residual powders produced at 220 °C for 12 h without stirring in the 5.0 M KOH solvent (12.5 mL) using different Si^4+^ precursor molar contents. Crystalline phases (▼) Ca_3_Cr_2_Si_3_O_12_ uvarovite structure, COD Card No. 96-900-7150.

Figure 4a shows typical PXRD patterns of Victorian green samples prepared at 220 °C for 12 h in the 5.0 M KOH solution without stirring. This experimental set aimed to investigate the effect of the nominal Si^4+^ content below the stoichiometric value of 3.0 mole required to produce the Ca_3_Cr_2_Si_3_O_12_. Crystallisation of the cubic structured Ca_3_Cr_2_Si_3_O_12_ occurred under alkaline hydrothermal conditions and was irrespective of the Si^4+^ deficiency induced in the reaction system. Thus, the Si^4+^ deficiency did not affect the equilibrium associated with the chemical reaction (Equation (2)) proposed for this reaction system that triggers the Ca_3_Cr_2_Si_3_O_12_ preparation by a single-step reaction. In addition, the nominal Si^4+^ deficiency did not produce any marked differences in the initial gel co-precipitation reaction. This inference is also supported by the dissolution of the Si-deficient precursor gel Ca:Cr:Si_x_, which was found to proceed rapidly as in the experiments conducted with the uvarovite precursor gel containing 3.0 mole of Si^4+^ described in Section 3.1. No secondary crystalline phases containing Ca^2+^ or Cr^3+^, namely calcium chromate, were produced during the experiments with the Si^4+^ molar deficiency (2.2–2.8 mole). Both metal ions were hydrolysed in the alkaline media as complex ions [16,17] (Equation (2)). The formation of hydro-garnet uvarovite on the residual powders prepared with various contents of Si^4+^ was confirmed by the FT-IR analyses, which are shown in Figure 4b. These results suggested that water absorption on uvarovite particles took place spontaneously during the particle crystallisation, even though the chemical equilibrium (Equation (2)) is reached with the Si^4+^ molar deficiency within the hydrothermal alkaline reaction media. These particular controlled set of experiments demonstrated that the Si^4+^ deficiency does not affect the Ca_3_Cr_2_Si_3_O_12_ synthesis, because the oxidation of Cr^3+^ to other metastable (Cr^4+^) or Cr^6+^ stable valences was hindered on the hydrothermal alkaline media.

Ca_3_Cr_2_Si_3−(y+x)_(OH)_24−(y+x)(gel)_ + ySiO_2(s)_ + 5NO_3_^−^_(aq)_ + 2Na^+^_(aq)_ → Ca_3_Cr_2_Si_3_O_12(s)_ + 3xCa(OH)_z_^n+^_(aq)_ + 2xCr(OH)_m_^n+^_(aq)_ + 5NO_3_^−^_(aq)_ + 2Na^+^_(aq)_ + 24 − (y + x) OH^−^_(aq)_ (2)

### 3.4. Morphology Evolution of Ca_3_Cr_2_Si_3_O_12_ Particles Prepared under Alkaline Hydrothermal Conditions

**Figure 5:**
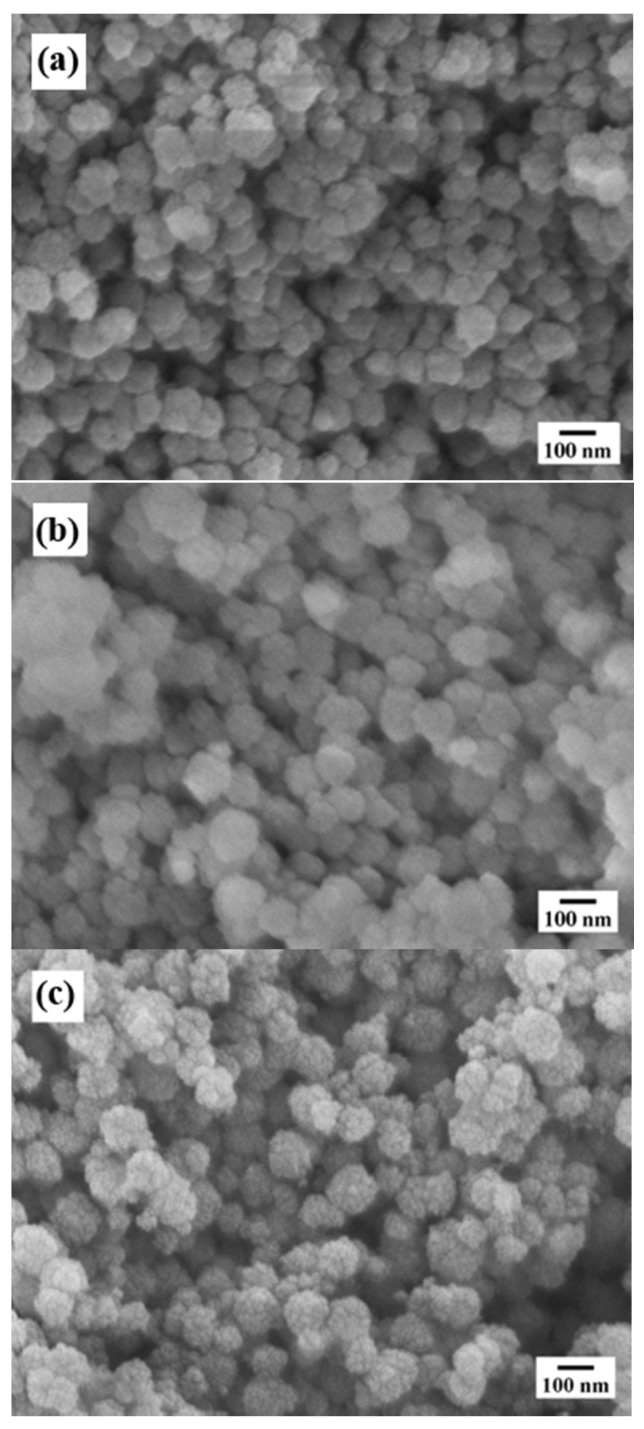
FE-SEM micrographs of Ca_3_Cr_2_Si_3_O_7_ powders produced under hydrothermal conditions at 220 °C for 24 h using a 5.0 M KOH solution (12.5 mL) and 3.0 mole Si^4+^ precursor mixed content; the experiments were conducted at a stirring speed (**a**) 50 rpm and (**b**) 0 rpm. (**c**) Micrograph of the powders prepared at 240 °C without autoclave stirring under the same experimental conditions.

The morphology and particle size of the Ca_3_Cr_2_Si_3_O_12_ particles synthesised at 220 °C for 24 h in a 5.0 M KOH solution with an Si^4+^ content of 3.0 mole, under both stirred (at 50 rpm) and static conditions, are shown in Figure 5a,b: respectively. SEM observations revealed that monodispersed Ca_3_Cr_2_Si_3_O_12_ agglomerates with a popcorn quasi-spherical shape were the dominant morphology under the alkaline hydrothermal conditions. These agglomerates are constituted of tiny anhedral crystals, which self-assembled to produce the 3D hierarchical “popcorn” architecture [20,21]. The anhedral particle size varies from 12 to 34 nm, as suggested by the crystallite sizes calculated from SEM micrographs (Figure 5a,b) and the results given in Table 1. Solvent convection provoked by the autoclave rotation limited the agglomerate growth. The popcorn agglomerate size produced with a 50 rpm rotation speed was 87 ± 17 nm. By way of contrast, coarser agglomerates up to 148 ± 3 nm were produced by maintaining the autoclave static inside the oven during the hydrothermal treatment (Figure 5b). Increasing the reaction temperature to 240 °C under static treatment conditions only resulted in a slight increase of the agglomerate size up to 156 ± 3 nm (Figure 5c). We surmise that a homogeneous colloid dispersion provoked by the autoclave agitation caused the reduction of the popcorn-shaped agglomerates. Agitation breaks up the colloid, accelerating its rapid dissolution in the solvent; as a consequence, a greater quantity of embryos is precipitated compared to that produced without stirring. The large molar volume of embryos homogeneously dispersed reduces the local solute saturation, hindering the particle coarsening [16,17]. Increasing the reaction temperature, without agitation, does not further coarsen the particles. The agglomerate growth was not significantly affected by increasing the temperature over 200 °C.

**Figure 6:**
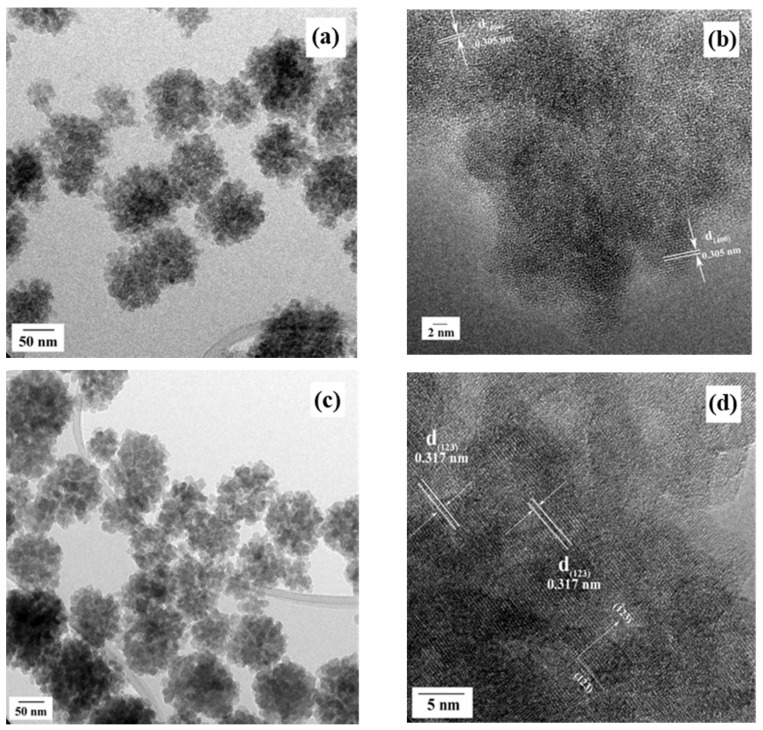
TEM (**a**,**c**) and HR-TEM (**b**,**d**) images of the popcorn shaped Ca_3_Cr_2_Si_2_O_12_ agglomerates hydrothermally prepared for 72 h without autoclave stirring and 3.0 mole Si^4+^ content using 12.5 mL of 5.0 M KOH as solvent. The one-pot hydrothermal treatment was carried at 200 (**a**,**b**) and 240 °C (**c**,**d**), respectively.

In addition, detailed crystalline structure features of the popcorn agglomerates were revealed by TEM observations. The analyses were conducted on residual powders prepared for 72 h at low (200 °C) and high (240 °C) temperatures; the TEM micrographs are shown in Figure 6. These images revealed that the bulk morphology of the popcorn-shaped agglomerates is irrespective of the treatment temperature. However, the size of the constituent anhedral particles was slightly increased from 8.5 ± 1.7 nm (200 °C) up to a mean size of 11.5 ± 2.0 nm by increasing the temperature to 240 °C. Details associated with the crystallinity and the self-assembly architecture of the fine anhedral Ca_3_Cr_2_Si_3_O_12_ particles were revealed by the HR-TEM observations (Figure 6b,d). Interestingly, these observations suggest that the anhedral particles produced at 200 °C exhibit a distorted atomic ordering. Nevertheless, some particles at the surface revealed that the assembly that took place in the basal plane with index (400), see Figure 6b. These Miller indexes correspond to an interplanar distance of 0.305 nm for the cubic garnet structure. On the contrary, at 240 °C, a remarkable atomic stacking occurred on the anhedral so that these particles exhibited a high crystallinity with the atomic ordering along a preferential direction indicated by the Miller index (123). This crystallographic plane was indexed with a calculated lattice fringe spacing of 0.317 nm, as portrayed in Figure 6d. Based on these interpretations, we argue that the reactivity of OH^−^ ions in the hydrothermal media caused a marked variation in the growth and the spontaneous self-assembling of the anhedral particles. Therefore, the dissolution–crystallisation mechanism reaction kinetics proceeded slowly at 200 °C, causing disruptions in the atomic stacking. The faster kinetics achieved at 240 °C caused a rapid solute supersaturation in the alkaline solvent, leading to correspondingly rapid embryo crystallisation and spontaneous epitaxial growth in the preferential crystallographic direction; this process is analogous to that recently determined for other silicate inorganic pigments [16,17]. This process, it is argued, controls the 3D hierarchical assembly, resulting in the formation of the Victorian green popcorn particles.

### 3.5. Diffuse Reflectance and Chromatic Properties of Ca_3_Cr_2_Si_3_O_12_ Victorian Green Powders

**Figure 7:**
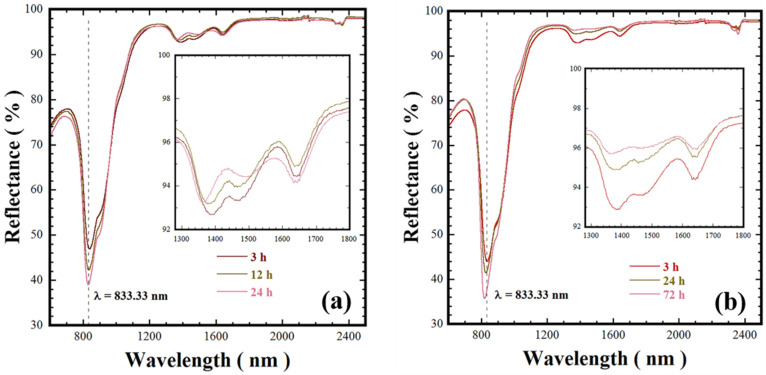
Near infrared reflectance spectra of the Victorian green pigments hydrothermally prepared in a 5.0 M KOH solution (12.5 mL) with 3.0 mole of Si^4+^ and no autoclave agitation. The powder pigments were synthesised at various reaction intervals and temperatures of (**a**) 200 and (**b**) 240 °C.

Near infrared to UV-vis diffuse reflectance analysis was carried out on the Ca_3_Cr_2_Si_3_O_12_ powders prepared at 200 and 240 °C and various reaction intervals without agitation. These samples produced with a stoichiometric Si^4+^ molar content (3.0 mole) exhibited differences in the particle size, green tonality, and structural crystallinity. The NIR reflectance data shown in Figure 7 were collected over wavelengths of 600–2500 nm using pelletised disks (10 mm diameter and 1 mm thickness). Generally, all the Victorian green powders prepared at 200 and 240 °C for both short and long reaction intervals exhibited an absorption peak at 833.33 nm. Furthermore, a marked increase in the reflectance took place in the NIR spectrum from 1000 and 2500 nm for all the samples, but all the pigments prepared at 200 °C (Figure 7a) had a slight reduction of 6% between 1300 and 1800 nm compared to those prepared at 240 °C. Interestingly, the pigment powders synthesised at 240 °C over a reaction interval of 24 h only had a tiny reflectance decay of approximately 2% below the maximum reflectance (98%) determined for powders produced over 72 h; see the inset in Figure 7b. These results, which were taken together with the crystalline structural differences revealed by HR-TEM, suggest that the uvarovite pigment NIR is maximised by improvement in the atomic stacking [22,23,24]. This is suggested to occur due to uvarovite dissolution–recrystallisation, which is promoted at reaction intervals over 24 h under hydrothermal conditions at 240 °C. It is worth mentioning that these results are especially relevant in highlighting a potential application for the prepared Victorian green pigments. In terms of energy, 52% of the sunlight reaches the Earth’s atmosphere falls within the spectrum of the NIR region (700–2500 nm). The incidence of this radiation on the surface of dark-coloured objects causes them to heat up. The reflectance behaviour of the uvarovite Ca_3_Cr_2_Si_3_O_12_ powders, as suggested by the NIR reflectance analyses of Figure 7, indicates that a potential application is as a “cold” pigment because it absorbs little NIR radiation. Such surface coatings could have a remarkable impact on energy-saving applications where solar radiation causes unwanted heating [24]. In general, bright colour TiO_2_ pigment has an 80% NIR radiation, resulting in a low heat up. This pigment is used to reduce the total solar energy absorbed. Furthermore, the Ca_3_Cr_2_Si_3_O_12_ powders have similar NIR reflectance properties to those submicron-sized blue pigments YIn_0.8_Mn_0.2_O_3_ [22] and YIn_0.9_Mn_0.1_O_3_-ZnO [24], which have a 90% near infrared reflectance property. Similarly, yellow BiVO_4_ pigments exhibit a reflectance above 80%, and the reflectance was maximised due to the BiVO_4_ polymorph formation provoked by the treatment temperature [25].

Victorian green powders colour characterisation was conducted using the CIE-L*a*b* colour space. The chroma value was calculated using the mathematical expression $C_{\mathrm{ab}}{}^{*}=\sqrt{{\left(a^{*}\right)}^{2}+{\left(b^{*}\right)}^{2}}$ (details reported elsewhere [25]). Table 2 summarises the chromatic coordinate L*a*b* and the chroma values determined for various pigments prepared under different conditions. The RGB colour coordinates obtained by transforming the L*a*b* values, and the pigment colour tonality associated with its RGB coordinates, are also given in Table 2. Generally, the results revealed that the powder colour is consistent with the standard CIELab coordinates of the Victorian green pigment. Although there were no significant variations in the chromatic values of single-phase uvarovite pigments, lighter bright green tonalities were associated with powders having chroma “C_ab_*” values within the range of 12.98–17.75. The pigments with chroma values ranging from 18.94 to 22.44 have a slightly darker green tonality. Based on these results, we surmise that the green tonality tuning is further enhanced by the microstructural parameters of the Ca_3_Cr_2_Si_3_O_12_ powders, particularly the popcorn-shaped agglomerate size and the refinement of the crystalline structure. This inference agrees with the experimental results obtained with the one-pot hydrothermal processing, because at temperatures over 220 °C in the alkaline medium (5.0 M KOH), well crystalline anhedral particles are produced, which are responsible for the variation on the Ca_3_Cr_2_Si_3_O_12_ green pigment hue.

The Si^4+^ deficiency did not affect the self-assembly process of the anhedral particles. However, based on the chemical equilibrium (Equation (2)), the hydrolysed metal cations reacted with the OH^−^, giving rise to the formation of complex hydroxide species (Ca(OH)_z_^n+^ and Cr(OH)_m_^n+^). This phenomenon caused a local reduction in the OH^−^ concentration, which slowed down the coarsening of the hierarchical 3D popcorn-shaped agglomerates, and this reaction took place using low Si^4+^ molar contents (2.2 and 2.6 mole). Hence, the particle size effect is the phenomenon responsible for trigger the bright green hue according to the natural light scattering mechanism. This mechanism is further enhanced in the nanometric-sized 3D popcorn-shaped agglomerates consisting of fine anhedral-shaped crystals (14.4–28.2 nm, see Table 1). Additionally, the dark green tonalities caused by the light interaction occur due to an increase in the crystallinity of the anhedral particles, together with the well-formed faceted surfaces (Figure 7a,b). Furthermore, the compact popcorn-shaped 3D arrangement could physically enhance the dynamic light reflection, giving the Ca_3_Cr_2_Si_3_O_12_ powders the properties of cool pigment, consequently reducing the light absorbance [4,11]. Hence, based on the optical measurements, we argue that the nano-sized hierarchical 3D popcorn-shaped Ca_3_Cr_2_Si_3_O_12_ agglomerate powders have potential application as cold pigments. Other applications include printing ink preparation, acrylic paints, and decorative purposes.

## 4. Conclusions

The synthesis of Victorian green pigment was successfully prepared in a highly concentrated KOH solution for the first time by the one-pot hydrothermal method. The nanosized aggregates crystallisation proceeded via a single-step chemical reaction that was achieved by the dissolution–crystallisation mechanism. The new processing approach is highly efficient, because the synthesis occurred even for a short reaction interval (3 h) at 200 °C without stirring. These conditions are adequate to synthesise nano-sized anhedral irregular particles; simultaneously, these particles underwent a spontaneous self-assembly that produce new nanometric 3D hierarchical popcorn-shaped agglomerates. Additionally, the colour tuning of the Ca_3_Cr_2_Si_3_O_12_ pigment was achievable with controlling the Si^4+^ deficiency (2.2–2.8 mole). The Ca_3_Cr_2_Si_3_O_12_ optical properties, colour hue, and NIR diffuse reflectance are affected by slight alterations in the particle size and crystallinity of the anhedral irregular particles forming the 3D hierarchical particle agglomerates. Hence, based on optical properties, the Victorian green powders prepared by the one-pot hydrothermal process have potential applications as a cold pigment source, and to prepare printing ink, acrylic paint, and other decorative purposes.

## Figures and Tables

**Table 1:** Summary of the relevant experiments selected to investigate the hydrothermal synthesis of single-phase Ca_3_Cr_2_Si_3_O_12_ particles and relevant Rietveld refinement results. The experiments were carried out with two volumes of the KOH solution 18.5 * and 12.5 ** mL.

Sample ID	KOH Solution [M]	Temperature (°C)	Time (h)	Stirring Speed (rpm)	Si^4+^ Nominal (mole)	Crystalline Phase	Phase Content (wt %)	Average Agglomerate Size (nm)	Rietveld Refinement Structural Parameters					
									Crystallite Size (nm)	“*a*_0_” (Å)	Cell Volume (Å^3^)	Lattice Strain	*R_wp_*	*GOF* (*χ*^2^)
CCS1	5.0 *	220	24	50	3.0	Uvarovite	100.0	-	18.91 (0.31)	12.3377 (63)	1878.06 (2.9)	0.49 (0.02)	7.14	2.97
CCS2	5.0 **	220	24	50	3.0	Uvarovite	100.0	87.0 ± 17.0	22.31 (1.29)	12.2579 (221)	1841.82 (9.9)	1.68 (0.05)	11.60	6.56
CCS4	2.5 *	220	24	50	3.0	Uvarovite	65.01	-	12.68 (0.46)	12.2379(18)	1832.82 (0.8)	1.05 (0.08)	6.73	2.43
						Quartz, SiO_2_	1.66							
						Ca_2_SiO_5_	16.93							
						Ca_2_SiO_4_	16.39							
CCS3	1.0 *	220	24	50	3.0	Quartz, SiO_2_	10.15	-	-	-	-	-	11.48	6.55
						CaCr_2_O_4_	41.84							
						Ca_2_SiO_4_	48.01							
CCS7	0.01 *	220	24	50	3.0	Amorphous	100.0	-	-	-	-	-	-	-
CCS8	0.0 *	220	24	50	3.0	Amorphous	100.0	-	-	-	-	-	-	-
CCS9	5.0 *	240	3	50	3.0	Uvarovite	100.0	-	22.65 (0.93)	12.3332 (148)	1876.00 (6.8)	0.76 (0.04)	13.62	7.25
CCS17	5.0 **	240	12	50	3.0	Uvarovite	100.0	145.0 ± 22.0	27.38 (1.56)	12.2468 (112)	1836.86 (5.1)	1.43 (0.04)	12.04	6.74
CCS19	5.0 **	240	6	0	3.0	Uvarovite	100.0	137.0 ± 25.0	55.86 (5.05)	12.2753 (157)	1849.71 (7.1)	1.85 (0.04)	14.48	6.83
CCS21	5.0 **	240	24	0	3.0	Uvarovite	100.0	156.0 ± 3.0	29.33 (2.24)	12.2575 (152)	1841.64 (4.8)	1.42 (0.03)	15.11	6.89
CCS23	5.0 **	240	72	0	3.0	Uvarovite	100.0	99.0 ± 20.0	23.87 (0.41)	12.2429 (39)	1835.08 (1.8)	0.78 (0.01)	6.96	2.82
CCS15	5.0 **	220	6	0	3.0	Uvarovite	100.0	173.0 ± 29.0	32.14 (4.93)	12.3016 (347)	1861.63 (15.7)	1.96 (0.10)	10.72	6.16
CCS14	5.0 **	220	12	0	3.0	Uvarovite	100.0	112.0 ± 15.0	19.96 (0.48)	12.2750 (87)	1849.55 (4.0)	1.05 (0.03)	6.90	2.84
CCS13	5.0 **	220	24	0	3.0	Uvarovite	100.0	148.0 ± 3.0	13.07 (0.25)	12.2475 (106)	1837.18 (4.8)	0.68 (0.05)	6.15	2.74
CCS31	5.0 **	220	12	0	2.6	Uvarovite	100.0	114.0 ± 17.0	14.45 (0.29)	12.2936 (67)	1858.00 (3.0)	0.49 (0.05)	6.20	2.79
CCS32	5.0 **	220	12	0	2.4	Uvarovite	100.0	104.0 ± 23.0	23.57 (1.13)	12.3277 (133)	1873.47 (6.0)	0.48 (0.04)	16.61	7.19
CCS33	5.0 **	220	12	0	2.2	Uvarovite	100.0	90.0 ± 28.0	28.26 (0.80)	12.3372 (75)	1877.82 (0.3)	0.73 (0.01)	6.51	2.88
CCS28	5.0 **	200	6	0	3.0	Uvarovite	100.0	-	12.79 (1.83)	12.3340 (984)	1876.35 (45.0)	2.0 (0.39)	12.52	5.88
CCS26	5.0 **	200	24	0	3.0	Uvarovite	100.0	-	12.66 (0.28)	12.2767 (136)	1850.33 (6.1)	0.88 (0.04)	6.63	2.76
CCS24	5.0 **	200	72	0	3.0	Uvarovite	100.0	66.0 ± 23.0	19.06 (0.44)	12.2708 (98)	1847.66 (4.4)	1.03 (0.03)	6.71	2.81

**Table 2:** Summaries of the particle sizes, CIE-L*a*b* values, chroma, RGB parameters, and colour of Ca_3_Cr_2_Si_3_O_12_ pigments prepared under stirred and static conditions; at different temperatures, reaction intervals, Si^4+^ deficiency with respect to the nominal concentration; and alkaline KOH media with different concentrations.

Sample ID	KOH Solution [M]	Temperature (°C)	Time (h)	Stirring Speed (RPM)	Si^4+^ Nominal (mole)	Average Agglomerate Size (nm)	CIELab Coordinates			RGB Colour Coordinates			Chroma C_ab_*	Colour Hue
							L*	a*	b*	R	G	B		
Gel CCS	-	-	-	-	-	-	64.31	−16.74	1.16	123	165	153	16.78	
CCS8	0.0	220	24	50	3.0	-	32.80	−43.49	14.88	0	93	51	45.97	
CCS7	0.01	220	24	50	3.0	-	41.75	−35.60	5.75	0	113	88	36.07	
CCS1	5.0	220	24	50	3.0	-	58.97	−17.78	−3.63	102	151	148	18.16	
CCS2	5.0	220	24	50	3.0	87.0 ± 17.0	58.03	−22.13	−3.66	88	151	145	22.44	
CCS4	2.5	220	24	50	3.0	-	67.04	−16.89	5.41	133	172	153	17.75	
CCS13	5.0	220	24	0	3.0	148.0 ± 3.0	59.98	−22.21	−4.24	93	156	151	22.62	
CCS21	5.0	240	24	0	3.0	156.0 ± 3.0	64.16	−18.75	−3.06	114	165	160	19.00	
CCS23	5.0	240	72	0	3.0	99.0 ± 20.0	62.99	−18.68	−3.10	111	162	157	18.94	
CCS24	5.0	200	72	0	3.0	66.0 ± 23.0	60.57	−20.04	−3.78	100	157	152	20.40	
CCS14	5.0	220	12	0	3.0	112.0 ± 15.0	66.52	−17.22	−1.78	125	171	164	17.31	
CCS31	5.0	220	12	0	2.6	114.0 ± 17.0	60.79	−19.48	−4.13	102	157	153	19.92	
CCS33	5.0	220	12	0	2.2	90.0 ± 28.0	72.44	−12.95	−0.79	151	185	179	12.98	

## Supplementary Materials

The following are available online at https://www.mdpi.com/2079-4991/11/2/521/s1, Figure S1: XRD diffraction results of treatments conducted at different temperature without stirring, Figure S2: The Rietveld refinement plots were calculated for the cubic Ca_3_Cr_2_Si_3_O_12_ and various secondary phases, Figure S3: FE-SEM micrographs of Victorian green pigments prepared with different Si^4+^ mole contents, Figure S4: DTA of Ca_3_Cr_2_Si_3_O_12_ prepared at various experimental conditions, Table S1: Summary of the chemical composition of the single-phase Ca_3_Cr_2_Si_3_O_12_, Tables S2–S7: CIF files of the crystalline phases determined in the XRD patterns of the powders prepared in hydrothermal conditions.
