# Supplementary material for: One-Pot Hydrothermal Synthesis of Victoria Green (Ca3Cr2Si3O12) Nanoparticles in Alkaline Fluids and Its Colour Hue Characterisation
Source: Nanomaterials (Basel). 2021 Feb 18;11(2):521. doi: 10.3390/nano11020521 (PMC7922063; doi:10.3390/nano11020521)
Supplement: Supplementary file 1 [file nanomaterials-11-00521-s001.zip › nanomaterials-1094176-supplementary.pdf]

Supplementary Materials

One-pot hydrothermal synthesis of Victorian green ($Ca_3Cr_2Si_3O_{12}$) nanoparticles in alkaline fluids and its colour hue characterisation

J. C. Rendón-Angeles[1*], Z. Matamoros-Veloza[2], J. L. Rodríguez-Galicia[1], G. Seong[3], K. Yanagisawa[4], A. Tamayo[5], J. R. Alonso[5], L. A. Anaya-Chavira[1]

[1]*Centre for Research and Advanced Studies of the National Polytechnic Institute, Saltillo Campus, Ramos Arizpe, 25900, Coahuila, México.*

[2]*Tecnológico Nacional de México /(I.T. Saltillo), Technological Institute of Saltillo, Graduate Division, Saltillo 25280, México.*

[3]*New Industry Creation Hatchery Center, Tohoku University, 6-6-10 Aoba, Aramaki, Aoba-ku, Sendai 980-8579, Japan*

[4]*Research Laboratory of Hydrothermal Chemistry, Faculty of Science, Kochi University, Kochi 780-8073*

[5]*Institute of Ceramics and Glass. CSIC. 28049 Madrid, Spain.*

The supporting file summarises details associated with some aspects related to the chemical reactions involved in the sol-gel coprecipitation for preparing the

S2.1. Chemical equations associated with the coprecipitation procedure for obtaining the precursor colloid suspension, the Ca:Cr:Si gels were subjected to the hydrothermal treatment.

Stoichiometric chemical reaction equilibrium.

$$3CaNO_{3(aq)} + 2Cr(NO_3)_{3(aq)} + 3Na_2SiO_{3(aq)} \xrightarrow{H_2O\ or\ KOH_{(aq)}}$$

$$Ca_3Cr_2Si_{3-x}(OH)_{24(gel)} + xSiO_{2(s)} + 5NO^-_{3(aq)} + 4Na^+_{(aq)} \qquad \text{Eq. (S1)}$$

The chemical reaction that takes into account the Si^{4+} content deficiency below the stoichiometric.

$$3CaNO_{3(aq)} + 2Cr(NO_3)_{2(aq)} + (3-x)Na_2SiO_{3(aq)} \xrightarrow{H_2O}$$

$$Ca_3Cr_2Si_{3-(x+y)}(OH)_{24-(x-y)(gel)} + 5NO^-_{3(aq)} + ySiO_{2(s)} + (3-x)Na^+_{(aq)} \qquad \text{Eq. (S2)}$$

S2.2. Characterisation of $Ca_3Cr_2Si_3O_{12}$ chemical composition

The details of the chemical route used to determine compositional aspects of the $Ca_3Cr_2Si_3O_{12}$ pigment powders are addressed in this document.

The element molar content in the $Ca_3Cr_2Si_3O_{12}$ compound was calculated using 20 mg ± 2 mg powder sample. The sample was preliminarily dissolved with 1 mL of hydrochloric acid (37 wt.%) at 30 °C using a Teflon beaker. After the powder dissolution, the liquor was diluted using a 1 M HNO_3 solution to a 50 ml in a volumetric flask. The chemical analyses were carried out with an ICP-AES, Shimadzu Multi-type I ICP-9000, Japan 6100. The ICP apparatus calibration was conducted using standard reagent analytical solutions (1000 mg/L) of each element. A specific volume of each standard solution of Ca, Cr and Si were diluted with a 1 M HNO_3 solution, to prepare standard solutions (100 ml) with the following concentrations: 1, 10 and 100 mg/L for each element. Calibration curves of Ca, Cr and Si, were plotted, and these were used to extrapolate the content of each metal cation in the solution. The results of selected $Ca_3Cr_2Si_3O_{12}$ Victorian green pigment chemical compositions are summarised in Table S1.

Table S1. Summary of the chemical composition of the single-phase $Ca_3Cr_2Si_3O_{12}$ Victorian green pigments prepared in a 5 M KOH solution under hydrothermal conditions, the experimental parameters studied were: the autoclave rotating speed, the nominal mole content of Si^{4+}, together with the reaction time and temperature.

Sample ID	Temperature (°C)	Time (h)	Stirring speed [RPM]	Si^{4+} molar content	Sample weight[a] (mg)	Sample weight[b] (mg)	Element chemical composition[c]			Elemental molar concentration Ca:Cr:Si[d]			Colour Hue[e]
							Ca (mg)	Cr (mg)	Si (mg)	Ca (mol)	Cr (mol)	Si (mol)	
CCS1	240	24	50		20.3	16.8	4.04 (0.02)	3.49 (0.01)	2.83 (0.02)	0.101 (2)	0.067 (1)	0.101 (2)	
CCS13	220	24			20.4	16.5	3.97 (0.02)	3.43 (0.01)	2.78 (0.02)	0.099 (2)	0.066 (1)	0.099 (2)	
CCS23	240	72		3.0	20.3	17.3	4.21 (0.02)	3.64 (0.01)	2.95 (0.02)	0.105 (2)	0.070 (1)	0.105 (2)	
CCS24	200	72			20.0	15.9	3.82 (0.02)	3.30 (0.01)	2.68 (0.02)	0.095 (2)	0.064 (1)	0.095 (2)	
CCS26	200	24	0.0		20.1	15.4	3.70 (0.02)	3.20 (0.01)	2.59 (0.02)	0.092 (2)	0.062 (1)	0.092 (2)	-
CCS14	220	12			20.1	16.7	4.01 (0.02)	3.47 (0.01)	2.81 (0.02)	0.100 (2)	0.067 (1)	0.100 (2)	
CCS31	220	12		2.6	20.3	16.2	3.89 (0.02)	3.37 (0.01)	2.73 (0.02)	0.097 (2)	0.065 (1)	0.097 (2)	
CCS33	220	12		2.2	20.1	16.4	3.94 (0.02)	3.41 (0.01)	2.76 (0.02)	0.098 (2)	0.066 (1)	0.098 (2)	

Note [a]Nominal weight of powder prior dissolution.

[b]Sample weight of the powder calculated using the weight of each element determined by ICP measurements.

[c]Weight of Ca, Cr and Si determined via ICP analyses; these results were used to calculate each sample elemental molar concentration. These concentrations fit the cation stoichiometric Ca:Cr:Si molar ratio of 3:2:3 corresponding to the Victorian green pigment.

[d]Elemental molar concentration determined for each powder sample using the elemental weight obtained by the ICP analyses.

[e]Colour hue determined from RGB coordinates in Table 2 in the manuscript.

S2.3. X-ray diffraction structural analysis of $Ca_3Cr_2Si_3O_{12}$ pigment powders.

These results address the analysis related to the effect of temperature and reaction interval, which were investigated under standard conditions of stoichiometric mixed Si^{4+} content of 3.0 mole and 5.0 M KOH solution without stirring, and these results are shown in Fig. S1. Fig. S1a shows typical XRD diffraction patterns of the powders obtained at 200 °C for various reaction intervals. The crystallisation of the $Ca_3Cr_2Si_3O_{12}$ crystalline phase as the main reaction product even occurred at reaction intervals as short as 3 h. The wet chemistry analyses of each pigment demonstrated that the single-phase uvarovite powders corresponded to the pure Victorian green pigment composition (Table S1). Furthermore, all the diffraction peaks in the 2θ range between 10–80° corresponding to the cubic uvarovite $Ca_3Cr_2Si_3O_{12}$, did not exhibit any marked displacements irrespective of the treatment reaction interval (3–72 h). Hence, we inferred that all the samples have the chemical composition of the Victorian green pigments.

A gradual change in the peak shape took place when the reaction interval extended over intermediate and long periods. The peaks became sharper-thinner for the pigment samples produced over 48 and 72 h at both at 200 ºC and 240 ºC (see Figs. S1a,b). XRD patterns exhibiting very tight peaks were produced even for shorter reaction intervals of 6–12 h at 240 ºC, as shown in Fig. S1b. The variations revealed in the X-ray diffraction against the temperature and reaction interval parameters might be associated with the structural differences in the crystallinity and particle size of the $Ca_3Cr_2Si_3O_{12}$ particles. This inference is supported by increasing the kinetic parameters (reaction interval or treatment temperature) leading to a structural variation in the resulting particles. Interestingly, these results demonstrate that the alkaline solution (5.0 M KOH) provokes a rapid dissolution of the precursor gel, as proposed by the reaction equilibrium of Eq. 1 even in a hydrothermal media without continuous stirring. Under these conditions, the nucleation and particle coarsening occurred at low temperature (200 °C) and over an interval as short as 3 h. These results proved that the colloid precursor reactivity, constituted by $Ca_3Cr_2Si_{3-x}(OH)_{24-4x}$ and SiO_2; is adequate in the 5.0 M KOH media to maintain the chemical equilibrium established by Eq. 1.

S2.4. Rietveld Refinement information and XRD results.

The cards of the crystalline phases determined in the reaction products XRD patterns are shown in Tables S2–S6, and the cards were taken from the COD 2014 database running in the High Score Plus Panalityca software 3.0e version. The crystallographic data was included in subroutines of the program algorithm designed to conduct the structural refinement. The algorithm is based in a 10-coefficient shifted Chebyshev polynomial function for modelling the background, and a pseudo-Voigt function fitted the profile peak shape. The refinement approach led to calculate the unit lattice cell parameters, the isotropic thermal displacement, the crystallite size and the secondary phase content.

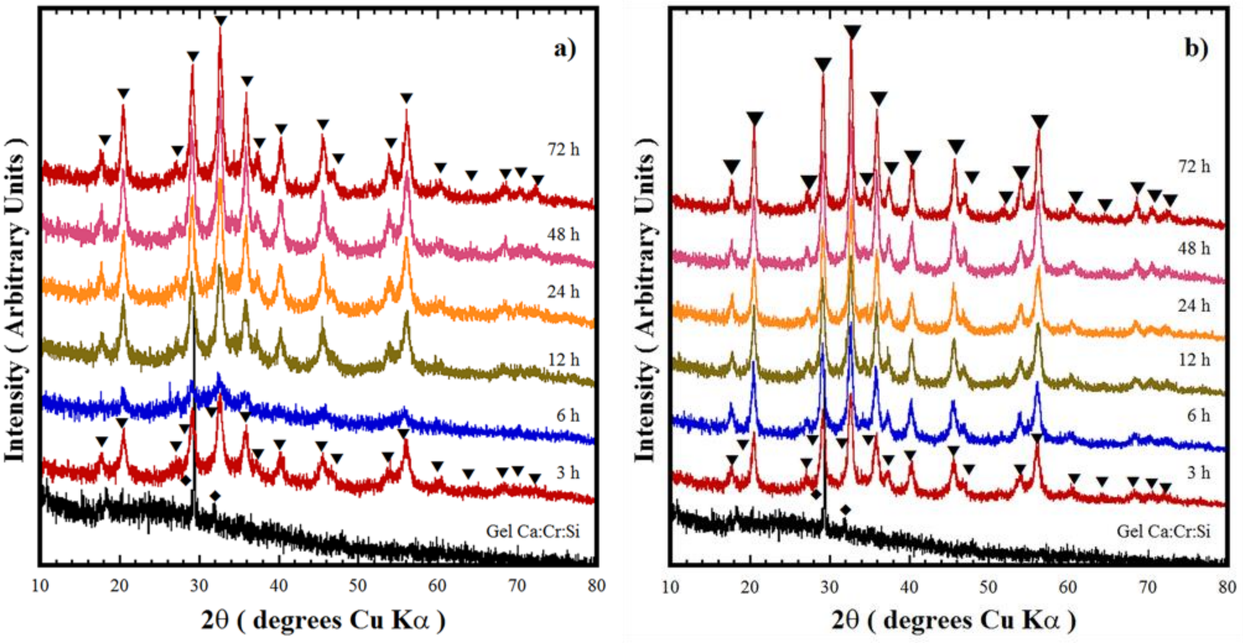

Figure S1. XRD patterns of $Ca_3Cr_2Si_3O_{12}$ samples hydrothermally prepared in a 5.0 M KOH solution with a Si^{4+} nominal 3.0 mole content at 200 °C (a) and 240 °C (b) for various reaction intervals, respectively. All the hydrothermal treatments were carried out at an autoclave filling ratio of 50% without stirring; the KOH solution volume used was 12.5 ml. Crystalline phase (▼) Uvarovite structure $Ca_3Cr_2Si_3O_{12}$, COD card no. 96-900-7150.

Table S2. The atomic coordinates of the $Ca_3Cr_2Si_3O_{12}$ with cubic structure (space group *Ia-3d*, *230*) used to carry out the Rietveld refinements by TOPAS 4.2 software; the spatial locations were reported previously elsewhere in the CIF file of the COD card no. 96-900-7150. And those of the secondary phases

**Element identification**	**Wyckoff position**	**Occupation**	**Spatial coordinates (** $Ca_3Cr_2Si_3O_{12}$ **)**		
			**x**	**y**	**z**
Ca	24c	1	0.1250	0.0000	0.2500
Cr	16a	1	0.0000	0.0000	0.0000
Si	24d	1	0.3750	0.0000	0.2500
O	96h	1	0.09567	0.2023	0.28917

Table S3. The atomic coordinates of the SiO_2, Quartz high hexagonal structure used to carry out the Rietveld refinements by TOPAS 4.2 software; the spatial locations were reported previously elsewhere in the CIF file COD card no. 96-900-5028.

**Element identification**	**Wyckoff position**	**Occupation**	**Spatial coordinates (** SiO_2 **)**		
			**X**	**y**	**z**
Si	3d	1	0.50000	0.0000	0.5000
O	6i	1	0.20765	0.4153	0.0000

Table S4. The atomic coordinates of the $CaCr_2O_4$, orthorhombic structure used to carry out the Rietveld refinements by TOPAS 4.2 software; the spatial locations were reported previously elsewhere in the CIF file COD card no. 96-200-2211.

Element identification	Wyckoff position	Occupation	Spatial coordinates ($CaCr_2O_4$)		
			X	y	z
Ca_1	2a	1	0.2500	0.2500	0.1481
Ca_2	2b	1	0.2500	0.7500	0.4648
Cr_1	4e	1	0.2500	0.0078	0.5058
Cr_2	4c	1	0.0000	0.0000	0.0000
O_1	4e	1	0.2500	0.0940	0.8380
O_2	4e	1	0.2500	0.5910	0.1790
O_3	8g	1	0.5030	0.0980	0.3300

Table S5. The atomic coordinates of the Ca_2SiO_4, monoclinic structure used to carry out the Rietveld refinements by TOPAS 4.2 software; the spatial locations were reported previously elsewhere in the CIF file COD card no. 96-210-3317.

Element identification	Wyckoff position	Occupation	Spatial coordinates (Ca_2SiO_4)		
			X	y	z
Ca_1	4e	1	0.3258	0.4210	0.0729
Ca_2	4e	1	0.1621	0.4181	0.4121
Ca_3	4e	1	0.1240	0.7889	0.4184
Ca_4	4e	1	0.4081	0.0681	0.2068
Si_1	4e	1	0.5221	0.2959	0.4326
Si_2	4e	1	0.0382	0.0757	0.2974
O_1	4e	1	0.3768	0.2007	0.0287
O_2	4e	1	0.4625	0.3647	0.2856
O_3	4e	1	0.5641	0.1350	0.4077
O_4	4e	1	0.6831	0.1127	0.0038
O_5	4e	1	0.0894	0.4557	0.1682
O_6	4e	1	0.2097	0.0091	0.3498
O_7	4e	1	0.0018	0.2130	0.3717
O_8	4e	1	0.0443	0.0955	0.1298

Table S6. The atomic coordinates of the SiO_2, Coesite monoclinic structure used to carry out the Rietveld refinements by TOPAS 4.2 software; the spatial locations were reported previously elsewhere in the CIF file COD card no. 96-901-2794.

Element identification	Wyckoff position	Occupation	Spatial coordinates (SiO_2)		
			x	y	z
Si_1	8f	1	0.18290	0.14110	0.07140
Si_2	8f	1	0.28480	0.09220	0.54260
O_1	4c	1	0.25000	0.25000	0.00000
O_2	4e	1	0.00000	0.36240	0.25000
O_3	8f	1	0.07590	0.12410	0.56540
O_4	8f	1	0.26220	0.14800	0.32660
O_5	8f	1	0.29720	0.03770	0.02550

Table S7. The atomic coordinates of the Ca_2SiO_5, orthorhombic structure used to carry out the Rietveld refinements by TOPAS 4.2 software; the spatial locations were reported previously elsewhere in the CIF file COD card no. 96-200-1356.

Element identification	Wyckoff position	Occupation	Spatial coordinates (Ca_2SiO_5)		
			X	y	z
Ca_1	4a	1	0.5797	0.2692	0.3160
Ca_2	4a	1	0.0812	0.7380	0.3214
Ca_3	4a	1	0.2434	0.0971	0.3614
Ca_4	4a	1	0.2547	0.4063	0.1393
Si_1	4a	1	0.4595	0.0813	0.0861
Si_2	4a	1	0.0338	0.4194	0.4060
O_1	4a	1	0.3824	0.1881	0.1844
O_2	4a	1	0.1063	0.3002	0.3157
O_3	4a	1	0.5987	0.0068	0.1765
O_4	4a	1	0.0928	0.0042	0.1764
O_5	4a	1	0.1456	0.0481	0.5550
O_6	4a	1	0.1541	0.5435	0.4368
O_7	4a	1	0.0400	0.3599	0.0312
O_8	4a	1	0.4560	0.1569	0.4718
O_9	4a	1	0.1992	0.6446	0.1412
O_10	4a	1	0.7042	0.3567	0.1491

Crystalline structural Rietveld refinement results:

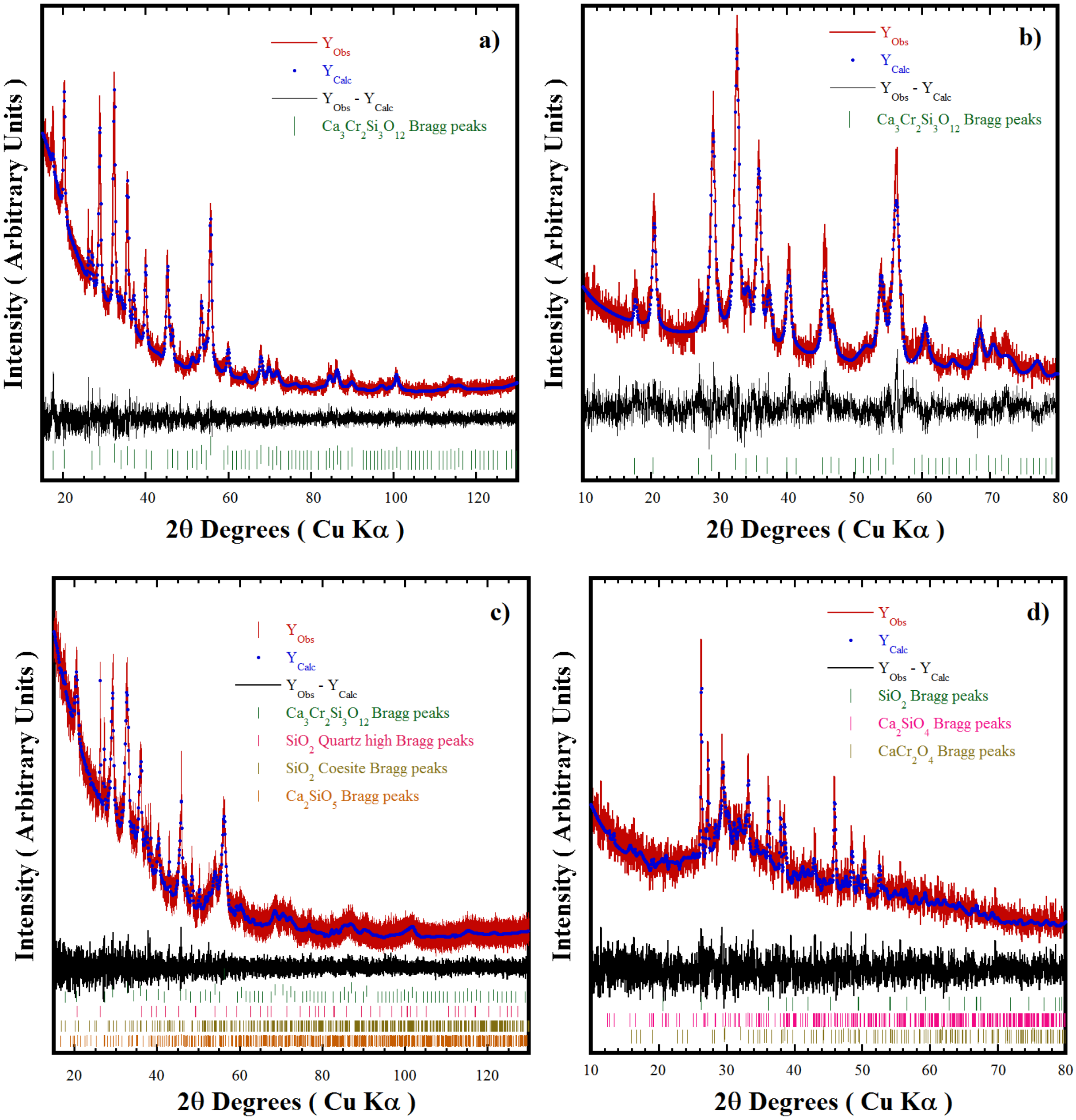

Figure S2. XRD patterns of $Ca_3Cr_2Si_3O_{12}$ sample prepared at 240 ºC for 24 h employing different concentration of the KOH solution (a,b) 5 M, c) 2.5 and d) 1.0 M under autoclave agitation speed of 50 RPM (a) and without agitation (b–d). The Rietveld refinement plots were calculated for the cubic $Ca_3Cr_2Si_3O_{12}$, space group *Ia-3d* (230), and the secondary phases were calculated according to the CIF files collected from the COD database.

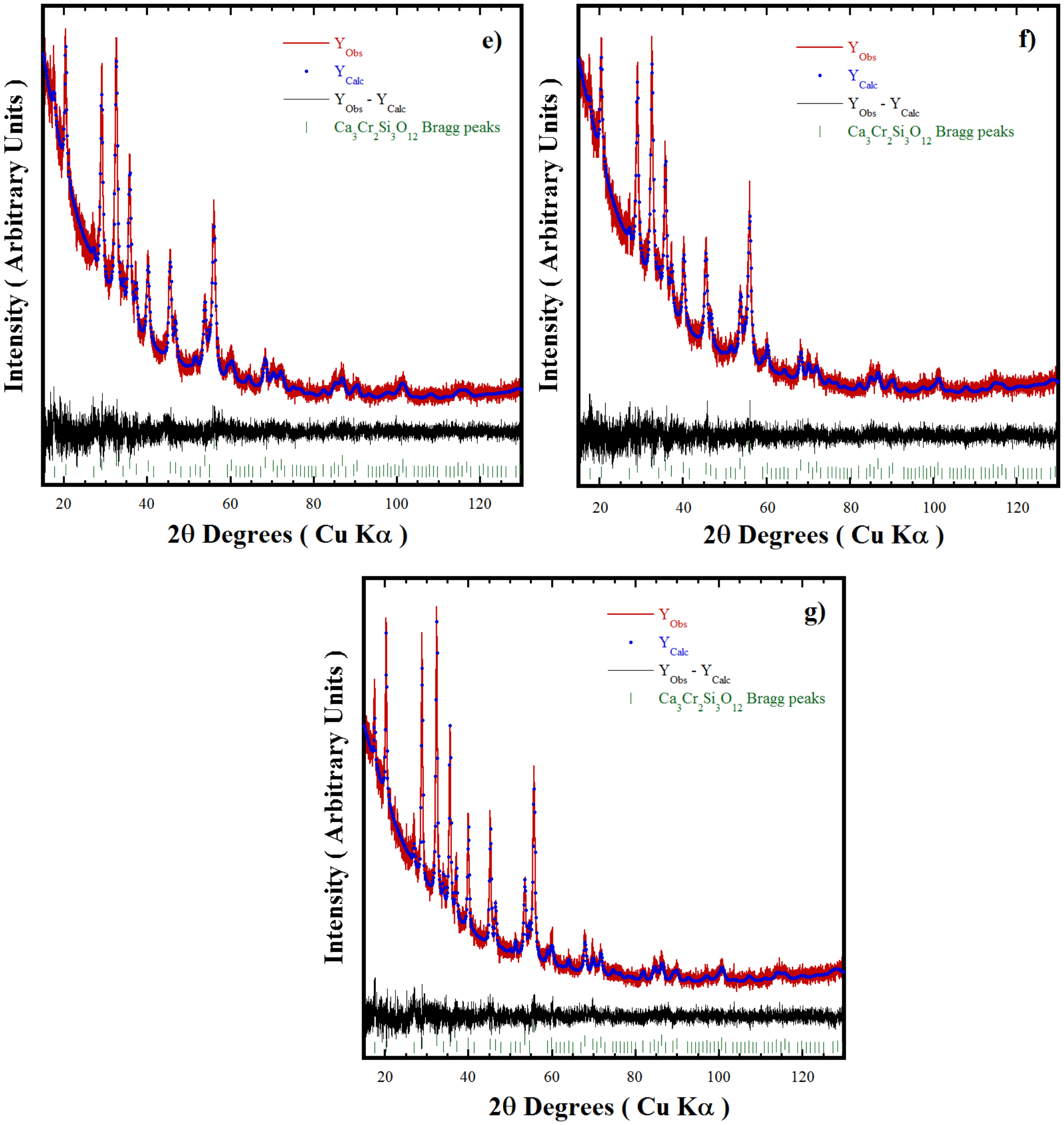

Figure S2 (Continue). XRD patterns of $Ca_3Cr_2Si_3O_{12}$ sample prepared at 220 ºC for 12 h in 5.0 M KOH solution (12.5 ml) without autoclave stirring. The powder pigments were prepared to employ different Si4+ nominal mole contents; e) 3.0, f) 2.6 g) 2.2 mole, respectively. The Rietveld refinement plots were calculated for the cubic $Ca_3Cr_2Si_3O_{12}$, space group *Ia-3d* (230).

S2.5. Microstructural analysis of the Victorian green pigments additional results.

Variation in the morphology of the 3D hierarchical popcorn-shaped agglomerates of $Ca_3Cr_2Si_3O_{12}$ was revealed when the synthesis was carried out using different nominal Si^{4+} contents varying from 2.2–3.0 mole; the changes are shown in Fig. 6. The SEM micrographs in Fig. S3 correspond to the Victorian green powders hydrothermally prepared at 220 °C for 12 h in a 5 M KOH solution without stirring using different contents of Si^{4+} (2.6, 2.4. and 2.2 mole). Agglomerates prepared with a Si^{4+}content of 2.8 mole, just below the stoichiometric level of 3.0 mole, did not affect the shape of the 3D hierarchical popcorn-like microstructure. However, using a Si^{4+} deficiency below 2.8 mole caused a marked reduction in the agglomerate size. Thus, the largest popcorn-shaped particles with a monomodal distribution; averaging 148 ± 3 nm, were preferentially synthesised employing 3.0 mole of Si^{4+}. In contrast, when Si^{4+} deficiencies of between 13.3 and 20 mol% (2.6 and 2.4 mole) were used, smaller particle sizes (114 ± 17 nm and 104 ± 28 nm) were produced, as seen in Figs. S3a, and S3b. The smallest particle sizes, with a mean value of 90 ± 28 nm, were obtained with a high Si^{4+} deficiency of 26.7 mol% (2.2 mole). However, these particles exhibit a bimodal distribution, as seen in the SEM micrograph in Fig. S3c. Under this concentration, the uvarovite 3D hierarchical particles had an average size of 90 ± 28 nm.

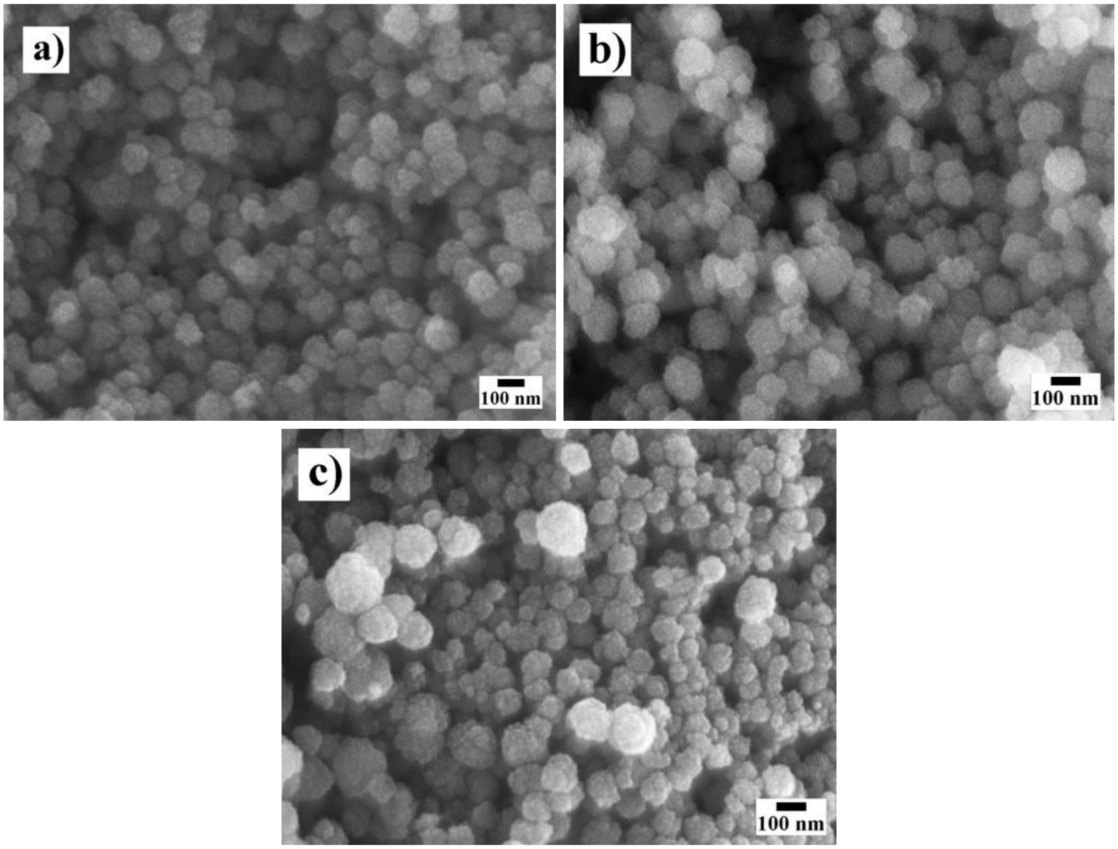

Figure S3. FE-SEM micrographs of Victorian green pigments $Ca_3Cr_2Si_2O_{12}$ produced under hydrothermal conditions at 220 °C for 12 h in a 5.0 M KOH (12.5 ml) solvent solution, and without autoclave stirring, the Si^{4+} precursor contents used were a) 2.6, b) 2.4, c) 2.2 mole, respectively.

The crystallite size determined by the Rietveld refinement analysis is likely associated with the anhedral particle size. This, together with the FE-SEM micrographs suggest that the number of anhedral particles was reduced due to the limited agglomerate coarsening promoted in solutions saturated with Ca^{2+} and Cr^{3+} cations [16]. The anhedral crystals underwent self-assembly that caused the development of popcorn-shaped agglomerates in all the cases studied (Fig. S3). We argue that the differences in the sizes of the popcorn-shaped agglomerates are provoked by Ca^{2+} and Cr^{3+} complex ions in the hydrothermal media. The content of these species within the colloid phase started to increase when stoichiometrically deficient Si^{4+} content of 2.2 mole is reached. We infer, based on our microstructural evaluation, that the excessive content of both metal cations does not alter the stoichiometric supersaturation state of the pigment constituents (Ca, Cr and Si) in the solvent, but it might accelerate the particle crystallisation kinetics and simultaneously hinders the particle growth. Likewise, an excess of the metal cations might gradually reduce the local concentration of OH^- ions required to minimise the solvent supersaturation level needed for decreasing the embryo molar volume crystallised, and the 3D hierarchical architecture assembly as well. Under reaction steady Si^{4+} deficient conditions the anhedral and agglomerate coarsening is limited, this inference is supported by the microstructure observations shown in Fig. S3.

S2.6. Thermal behaviour of the ***$Ca_3Cr_2Si_3O_{12}$ particles hydrothermally produced in mild alkaline hydrothermal reaction media.***

The thermal stability of the $Ca_3Cr_2Si_2O_{12}$ powders prepared at 240 ºC for with a 5 M KOH using 3.0 mole of Si^{4+} is given by the differential thermal analyses shown in Fig. S2. Fig. S2 includes the thermograph and weight loss (wt %) of the precursor Ca:Cr:Si gel, this sample exhibited two events revealed at temperatures of 222 and 488 ºC, of exothermic and endothermic nature; respectively. These events might be attributed to the water weight loss of 20 wt.% caused by water vapour release at 200 ºC. The tiny increase in weight between 450–490 ºC, is argued to the amorphous compound crystallisation. In particular, according to the total weight loss determined on the precursor Ca:Cr:Si gel, water corresponding to the OH^- ions is approximately 38.5 wt% of the total amount. Above 500 ºC the precursor gel showed a similar thermally stability, as revealed for the hydrothermally prepared Victorian green pigments up to 1000 ºC.

On the other hand, all the pigment samples prepared at different experimental conditions did not exhibit endothermal or exothermal events associated with pigment chemical decomposition due to thermal exposure. However, the sample weight loss occurred in two steps within the temperature interval of 100–500 ºC. Above 500 ºC, only slight variations (± 3.0 wt%) were determined, thereby the total weight loss calculated approximately was 17.0 ± 2.5 wt%. These results agree with the unit cell lattice parameters and the residual water molecule content determined by the FT-IR analyses. Generally, the pigments DTA curve showed a marked decrease up to 90 ºC; above this temperature, the baseline corresponding to the three hydrothermally prepared powders gradually decreased until 1000 ºC. According with the thermal analyses, we surmise that the present results demonstrate that these powder pigments are likely to have a potential application in the preparation of acrylic paints within the green colour Pantone spectrum, or the colouring of polymer substrates. The possible usage

of Victorian green pigment in the preparation of ceramic colour enamels can be carried out by heat treatment of the prepared $Ca_3Cr_2Si_3O_{12}$ pigments to eliminate the water absorbed.

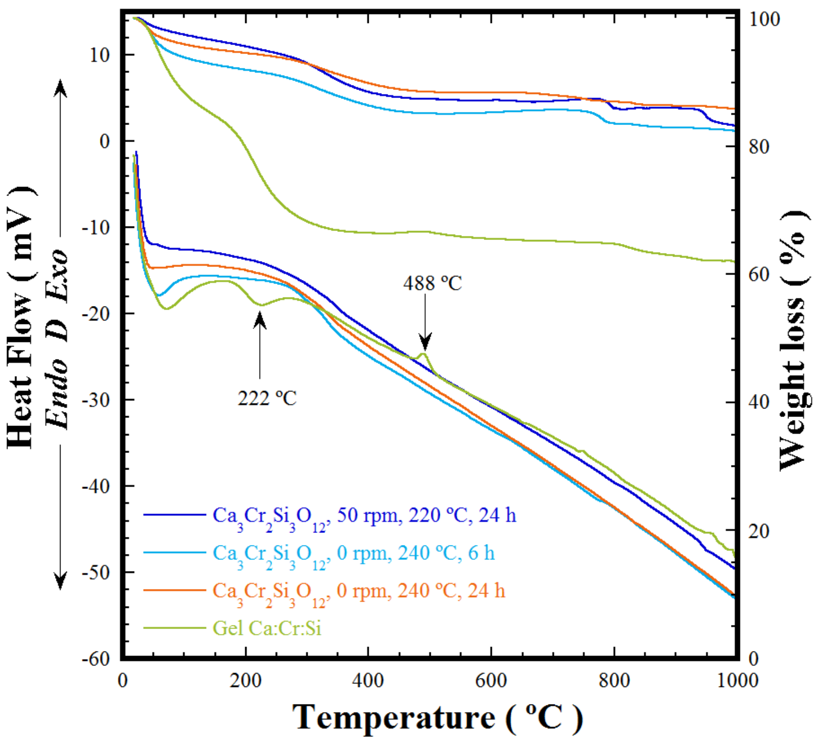

Figure S4. Curves corresponding to differential thermal analysis of $Ca_3Cr_2Si_3O_{12}$ samples hydrothermally prepared using 12.5 ml of 5.0 M KOH solution and a nominal molar Si^{4+} content of 3.0 mole different reaction temperature and time; these analyses were carried out up to 1000 ºC in air atmosphere.
